# Characterization of nanoparticles and fluorescent recombinant extracellular vesicles using three different generations of high-sensitivity flow cytometers

**DOI:** 10.20517/evcna.2026.33

**Published:** 2026-08-18

**Authors:** Estefanía Lozano-Andrés, Ye Tian, Sten F.W.M. Libregts, An Hendrix, Xiaomei Yan, Ger J.A. Arkesteijn, Marca H.M. Wauben

**Affiliations:** ^1^Division of Cell Biology, Metabolism and Cancer, Department of Biomolecular Health Sciences, Faculty of Veterinary Medicine, Utrecht University, Utrecht 3584 CM, The Netherlands.; ^2^Division of Infection & Immunology, Department of Biomolecular Health Sciences, Faculty of Veterinary Medicine, Utrecht University, Utrecht 3584 CL, The Netherlands.; ^3^MOE Key Laboratory of Spectrochemical Analysis & Instrumentation, Key Laboratory for Chemical Biology of Fujian Province, State Key Laboratory of Physical Chemistry of Solid Surfaces, Department of Chemical Biology, College of Chemistry and Chemical Engineering, Xiamen University, Xiamen 361005, Fujian, China.; ^4^Laboratory of Experimental Cancer Research, Department of Human Structure and Repair, Ghent University, Ghent 9000, Belgium.; ^5^Cancer Research Institute Ghent, Ghent 9000, Belgium.

**Keywords:** Extracellular vesicles, exosomes, high-sensitivity, flow cytometry, silica nanoparticles, characterization, cross-platform, reference material

## Abstract

**Aim:** High-sensitivity flow cytometry (FC) allows multiparametric analysis of nanoparticles (NPs) and extracellular vesicles (EVs). With new instruments available, studies that evaluate their performance using the same materials in a controlled environment are required. Here, we performed a comparative study to investigate the capabilities of three flow cytometers, the NanoFCM (NF), BD Influx (IF) and CytoFLEX LX (CF).

**Methods:** Firstly, we analyzed a mixed population of silica NPs (SiNPs, 68, 91, 113 and 155 nm) by using light-scatter-based detection thresholds [side scatter (SSC), forward scatter (FSC), violet side scatter (VSSC)] across a concentration range from 10^6^ to 10^9^ particles/mL. Next, we analyzed fluorescent recombinant EVs (rEVs) by comparing light-scatter-based thresholding (488 nm SSC available for all platforms), the combination of SSC thresholding with a fluorescent gate, and fluorescent thresholding.

**Results:** Upon qualitative and quantitative analysis, we observed that instruments differed in sensitivity, the NF could detect 68 nm SiNPs, while both IF and CF were able to detect down to 91 nm SiNPs when using a scatter-based threshold, which was improved by using FSC and VSSC compared to SSC, respectively. We show that the NF required a higher sample concentration to ensure optimal detection, while IF and CF benefited from more diluted samples. Next, we defined a single particle detection range measuring fluorescent rEV and demonstrated that fluorescence-based detection improved the detection of particles of interest due to lower background interference.

**Conclusion:** We here provide the strengths and limitations for each platform regarding the analysis of differently sized NPs at different sample concentrations.

## INTRODUCTION

Flow cytometry (FC) has become a powerful nanotechnology for high-throughput analysis of single nanoparticles (NPs) and extracellular vesicles (EVs). It allows the qualitative and quantitative characterization of heterogeneous cell-derived EV samples as long as the specific requirements are met^[1,2]^. Traditionally, the forward scatter (FSC) parameter in FC is used to define the minimum signal required to detect an event, also known as the threshold (t). For cells, this threshold is typically set well above the background noise of the instrument. However, in the case of EVs, which are orders of magnitude smaller than cells and scatter considerably less light, the threshold often approaches the instrument's noise level, posing significant challenges for their reliable detection^[3]^. In the past decade, diverse FC approaches have been proposed for the characterization of such 4-sized particles^[2]^. First reports of single EV FC analysis utilized optimized and tailored first-generation FC instruments and employed a fluorescence-based detection strategy, requiring all events of interest to be sufficiently bright to be detected^[4]^. A second generation of instruments that collect light scattering with higher sensitivity [violet 405 nm laser side scatter (VSSC)] and accommodate both cellular and small particle measurements made the use of FC in the EV field more accessible^[1,5]^. These second-generation flow cytometers are often equipped with enhanced sensitivity features, e.g., a small particle detector combined with VSSC or the 488 nm laser side scatter (SSC), allowing a single light scatter parameter to be used for EV detection. In parallel, a third generation of instruments with a bottom-up design fully dedicated to nanoparticle analysis was initiated with a state-of-the-art, lab-built nanoflow cytometer. This instrument utilizes an SSC parameter from the 532 nm laser, achieving a sizing resolution comparable to cryogenic electron microscopy (cryo-EM) for EV characterization^[6,7]^. The technology has since been commercialized by NanoFCM Inc., demonstrating broad utility across diverse applications^[8,9]^.

Despite these advancements, analyzing the whole size range of EVs at the single-particle level remains challenging due to their great heterogeneity, highly variable concentrations, and signals that are frequently close to, at, and/or below the limit of detection (LOD) of the instrument. Previous studies often used polystyrene (PS) NPs to report the LOD. However, the refractive index (RI) of PS is higher than that expected from EVs, resulting in polystyrene nanoparticles (PSNPs) scattering one to two orders of magnitude more light than EVs of a similar size^[3]^. Alternative materials with a lower RI, such as silica nanoparticles (SiNPs) are thus essential for instrument characterization when unknown samples of low RI will be analyzed. Such SiNPs have been used on dedicated third-generation instruments to report size estimations of EVs based on Mie theory modelling^[6,10]^.

Besides synthetic NPs, reference materials (RM) that are carefully designed to resemble biological EV samples will further aid the validation of flow cytometers able to detect and quantify single particles with high sensitivity^[10]^. Stable fluorescent biological recombinant EVs (rEVs) and synthetic EV-mimetics composed of a lipid bilayer and canonical EV markers with an average diameter of 125 nm and RI of 1.39 are described^[11-13]^. Currently, the rEVs developed by Geeurickx *et al*. are commercially available under the name of exosome standards, fluorescent (Sigma-Aldrich)^[13]^.

In this comparative study, we aim to evaluate and compare the detection of mixed populations of SiNPs and fluorescent rEVs across three generations of flow cytometers, a fully tailored first generation optimized BD Influx^TM^ (IF), a commercial second generation CytoFLEX LX^TM^ (CF) and a state-of-the-art third generation NanoFCM^TM^ (NF), by measuring the same samples simultaneously on each platform in the same location by dedicated operators using identical instrument settings. Here we identified the limitations of various detection strategies based on light scatter and fluorescence thresholding. Moreover, we show how sample concentration impacts the detection of single particles on each system when improved thresholding is applied. Using fluorescent rEVs, we further demonstrate the value of well-characterized RM and the use of fluorescent signals for comparing instrument performance and achieving reliable particle quantification.

## METHODS

### SiNPs

The Silica Nanospheres Cocktail (S16M-Exo, NanoFCM), a mixture of 68, 91, 113, and 155 nm SiNPs, was provided by NanoFCM Inc. The SiNP samples were diluted in Milli-Q H_2_O (filtered 0.22 μm, Merck) prior to the measurements.

### Recombinant fluorescent EVs (rEVs)

Commercially available fluorescent rEVs expressing green fluorescent protein (GFP) (SAE0193-1VL, Sigma Aldrich) were used. Production and characterization have been previously described in detail^[13]^. Dilutions to obtain the desired concentrations were made in commercial phosphate-buffered saline (PBS) (Corning) shortly before measurements. The stock concentration used for these experiments was determined by fluorescent Nanoparticle Tracking Analysis (NTA) to be 1.35E + 13 fluorescent particles/mL.

### Setup of flow cytometer platforms

#### NanoFCM (NF)

The NanoFCM N30 model (NanoFCM Inc., Xiamen, China), referred to as NF, was equipped with a 488 nm laser line and single-photon counting module (SPCM) detectors. For this study, we used an SSC threshold (488 nm laser) at 40 mW and 10% of SSC decay, following the manufacturer’s instructions. The SSC signal and fluorescence collection were performed using 488/10 and 525/40 nm band-pass filters (central wavelength/bandwidth), respectively. The threshold levels for both the peak height (a digital discriminator set to 3 times the standard deviation of the background) and the peak width (0.3 ms) were used as criteria for burst (or peak) identiﬁcation. Optical alignment was standardized using a reference material [200 nm PS quality control (QC) beads, NanoFCM]. The sample was boosted into the flow cytometer for 1 min before data acquisition. To ensure no cross-contamination between samples, the sample injection capillary was rinsed with washing solution (NanoFCM) for 1 min before each sample loading. Low sample flow rate for the measurements was calibrated as 0.005 μL/min. All samples were recorded for a fixed time of 120 s.

#### BD Influx (IF)

The jet in air-based BD Influx (BD Biosciences, San Jose, CA), referred to as IF, was equipped with 488, 405, 561, and 635 nm laser lines and photomultiplier tube (PMT) detectors. An improved reduced-wide-angle FSC (rw-FSC) detection was used^[14]^, by implementing an 8-mm obscuration bar and a 200-μm pinhole combination. Further details about its configuration and adaptations are described elsewhere^[4]^. The band-pass fluorescence collection filter used was 530/40 nm. Low sample flow rate was calibrated as 6 μL/min.

Upon acquisition, all scatter and fluorescence parameters were set to a logarithmic scale. To ensure that each measurement was comparable, a workspace with predefined gates and optimal PMT settings for the detection of 100 and 200 nm yellow-green (505/515) FluoSphere beads (Invitrogen, F8803 and F8848) was loaded. Upon aligning the fluid stream and lasers the 100 and 200 nm bead populations had to meet the criteria of pre-defined MFI and scatter values within these gates, where they displayed the smallest coefficient of variation (CV) for SSC, reduced wide-angle forward scatter (rw-FSC) and Fluorescence channel 1 (FL-1). The trigger threshold levels were set by running a buffer control sample next to the SiNP sample, thereby allowing the lowest possible threshold value without compromising detection sensitivity. The selected trigger values were SSC threshold [488 nm laser at 0.32, arbitrary units (a.u.)], rw-FSC (488 nm laser at 0.29, a.u.) and FL (488 nm laser at 0.61, a.u.).

Upon loading the sample, the sample was boosted into the flow cytometer until events appeared, after which the system was allowed to stabilize for 30 s. All samples were then recorded for a fixed time of 120 s using BD FACS Sortware 1.01.654 (BD Biosciences). Between measurements of samples, the sample line was washed subsequently with BD FACSRinse (BD Biosciences) and the dilution buffer for 5 s.

#### CytoFLEX LX (CF)

The CytoFLEX LX (Beckman Coulter, Brea, CA), referred to as CF, with a cuvette-based system, was equipped with 375, 405, 488, 561, 638, and 808 nm laser lines and avalanche photodiode (APD) detectors. We performed QC according to the manufacturer’s instructions. Upon successful QC, we measured 100 and 200 nm yellow-green (505/515) FluoSphere beads (Invitrogen, F8803 and F8848) to validate pre-defined gate positions for each using adjusted gain values for SSC (488 nm), VSSC (405 nm) and FL (488 nm). All gain and threshold values were kept constant during the study. Specifically, the SSC threshold (488 nm laser) was set at 500 a.u., the VSSC threshold (405 nm laser) was set at 1,600 a.u. and FL threshold (488 nm laser) at 600 a.u. The band-pass fluorescence collection filter used was 525/40 nm. Low sample flow rate was estimated to be 10 μL/min. Upon loading the sample, we allowed the system to stabilize for 30 s before recording. All samples were recorded for a fixed time of 120 s. Backflush was performed in between samples to minimize sample carryover. Data files were acquired with CytExpert Software (Beckman Coulter) and exported for further analysis.

When performing quantitative and qualitative analysis of particles, sample dilutions were performed in each buffer (either filtered Milli-Q H_2_O or commercial PBS, as indicated in the legends of Figures 1-4). Detailed descriptions of each instrument and methods are provided in Supplementary Table 1 (MIFlowCyt checklist) and Supplementary Table 2 (MiFlowCyt-EV framework). Data analysis was performed in FlowJo Version 10.5.0. Data were handled in Microsoft Excel, and figures were prepared using GraphPad Prism version 10.0 (GraphPad Software Inc) and Adobe Illustrator (V28.0, Adobe Inc).

**Figure 1:**
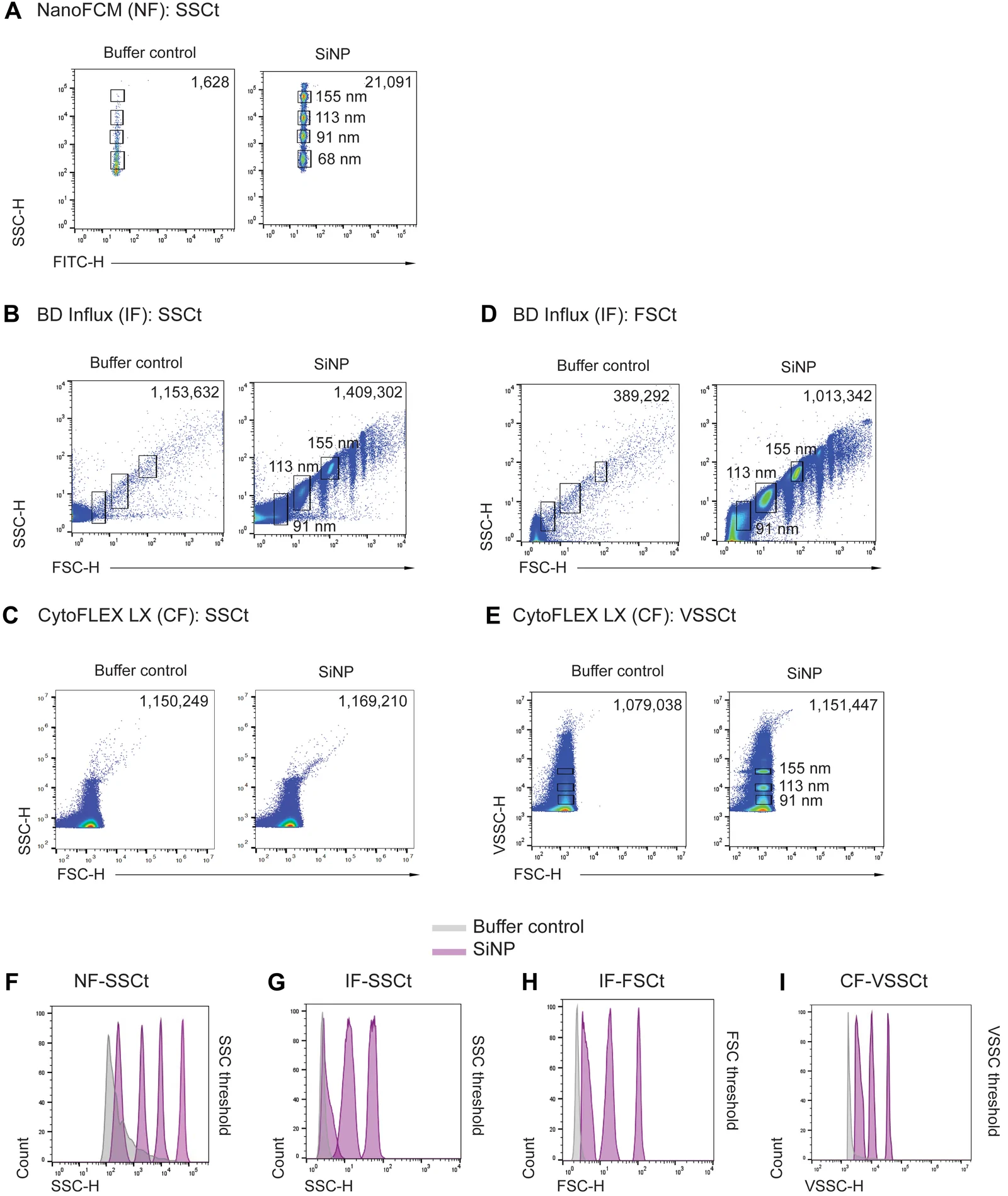
Light scatter-based analysis of buffer control and a mixed population of non-fluorescent SiNPs across platforms. Dot plots displaying the number of events as indicated in buffer control and the sample containing four size-defined populations of non-fluorescent SiNPs (68, 91, 113, 155 nm in diameter). Samples were measured on the NanoFCM with (A) an SSC (488 nm) threshold, on the IF with (B) an SSC (488 nm), on the CF with (C) an SSC (488 nm), on the IF with (D) an rw-FSC (488 nm) threshold, and on the CF with (E) a VSSC (405 nm) threshold. SiNP samples were pre-diluted shortly before measurements (1:100 for NF and 1:1,000 for both IF and CF). Samples were first gated to minimize the inclusion of doublets and aggregates (shown in Supplementary Figure 1). Next, each SiNP subset was gated, and the respective size is indicated within the plot; (F-I) Overlay histograms showing buffer control and gated SiNP subsets displaying each indicated scatter parameter. Identical buffer control and SiNP samples were acquired simultaneously on all three instruments in the same room under the same conditions. The figure shows data obtained from one selected sample dilution measured on each instrument with the indicated threshold (SSCt on NF, SSCt and FSCt on IF, and SSCt and VSSCt on CF). In total, 6 serial sample dilutions from the same stock solution were measured on the NF, while 12 serial dilutions were measured on the IF and the CF. CF: CytoFLEX LX; IF: BD Influx; NF: NanoFCM; FSC: forward scatter; rw-FSC: reduced wide-angle forward scatter; FSCt: forward scatter threshold; SSC: side scatter; SSCt: side scatter threshold; VSSC: violet side scatter; VSSCt: violet side scatter threshold; SiNPs: silica nanoparticles.

**Figure 2:**
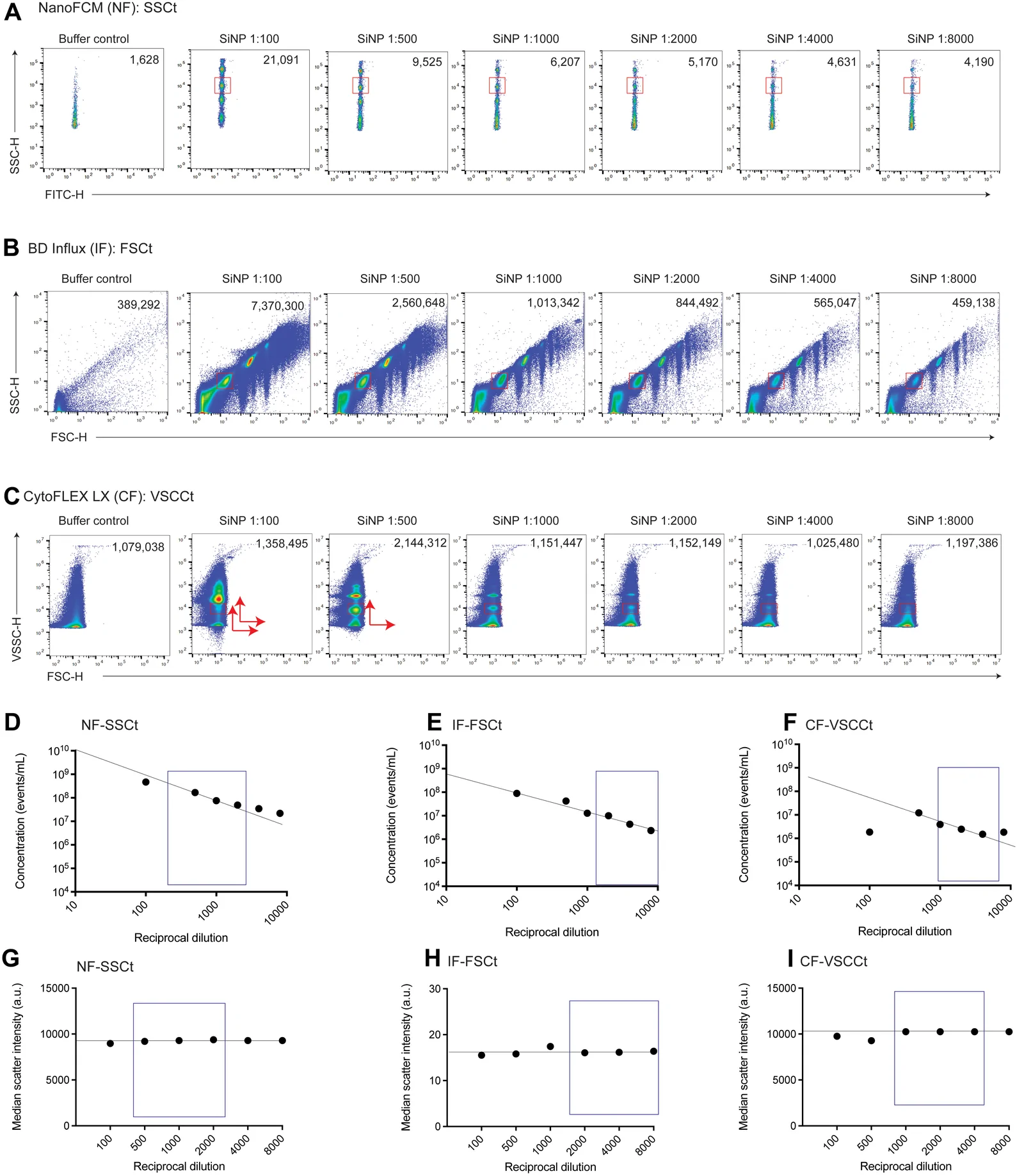
Analysis of six sample dilutions from non-fluorescent SiNPs across platforms. Dot plots displaying SSC-H *vs*. FITC-H or FSC-H from the total number of events of buffer control (left panel) and non-fluorescent SiNPs measured for 2 min (A) on the NF with an SSC (488 nm) threshold, (B) on the IF with an rw-FSC (488 nm) threshold, and (C) on the CF with a VSSC (405 nm) threshold. First, events were gated to minimize the inclusion of aggregates and doublets after inspection of area, height, or width parameters [Supplementary Figure 1]. Second, the indicated gate (red) was used to select the 113 nm SiNP population across dilutions and instruments. Note that the CF SiNP 1:100 and 1:500 samples were too concentrated to adequately gate around this population (red arrows). Samples were diluted as indicated in the top row (from 1:100, 1:500, 1:1,000, 1:2,000, 1:4,000, and 1:8,000, left to right); (D-F) Concentration of detected events across dilutions was calculated for each 113 nm gated sample following normalization for each instrument flow rate; (G-I) Median scatter intensity values (a.u.) from the gated 113 nm population were plotted for the NF, IF, and CF, respectively, for each diluted sample. The blue box indicates the samples that followed the expected event reduction trend while keeping a constant scattering intensity for this population. The figure shows data obtained from 6 measured samples originating from the same stock solution. NF: NanoFCM; F: BD Influx; CF: CytoFLEX LX; FITC: fluorescein isothiocyanate; FSC: forward scatter; FSC-H: forward scatter height; rw-FSC: reduced wide-angle forward scatter; SSC: side scatter; SSC-H: side scatter height; VSSC: violet side scatter; VSSC-H: violet side scatter height; SiNPs: silica nanoparticles; a.u.: arbitrary units.

**Figure 3:**
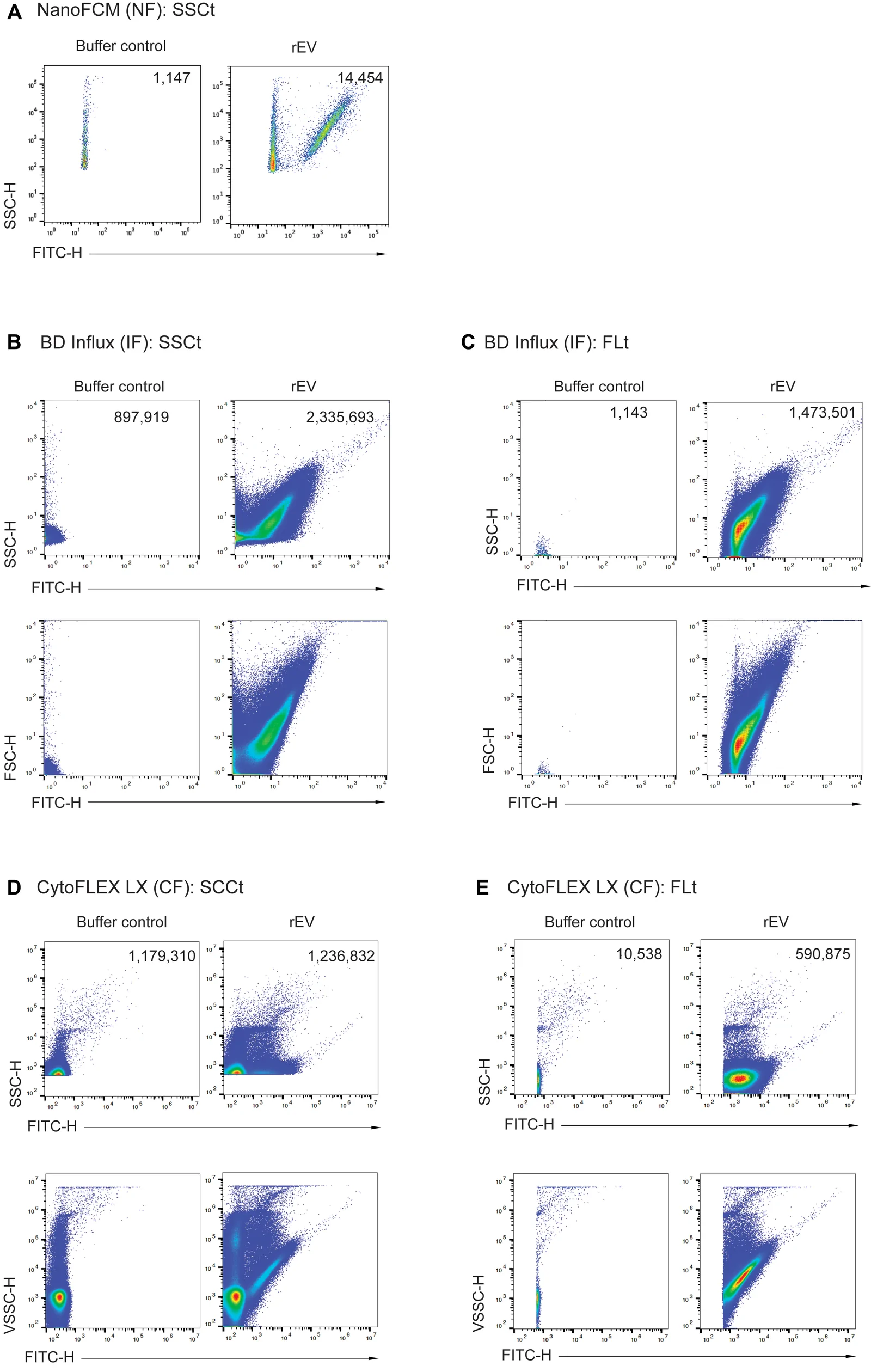
Qualitative analysis of fluorescent rEVs across different platforms. Dot plots from the PBS buffer control, here used as dilution buffer (left), and fluorescent rEVs (right) are shown for eGFP fluorescence (measured under FITC-H) *vs*. light scattering (either SSC-H, FSC-H, or VSSC-H) as indicated for each plot. Measurements (A) on the NF with an SSC threshold; (B) on the IF with an SSC threshold; (C) on the IF with an FL threshold; (D) on the CF with an SSC threshold; and (E) on the CF with an FL threshold. Total number of events measured is indicated in each dot plot. The rEV samples were pre-diluted before measurements; dot plots shown correspond to a 1:1,000 dilution for NF and a 1:32,000 dilution for both IF and CF. The data shown is representative of two independently performed experiments acquiring 6 diluted samples from the same stock on each instrument. NF: NanoFCM; IF: BD Influx; CF: CytoFLEX LX; SSC: side scatter; SSC-H: side scatter height; VSSC-H: violet side scatter height; eGFP: enhanced green fluorescent protein; FL: fluorescence; FSC-H: forward scatter height; PBS: phosphate-buffered saline; rEVs: recombinant extracellular vesicles.

**Figure 4:**
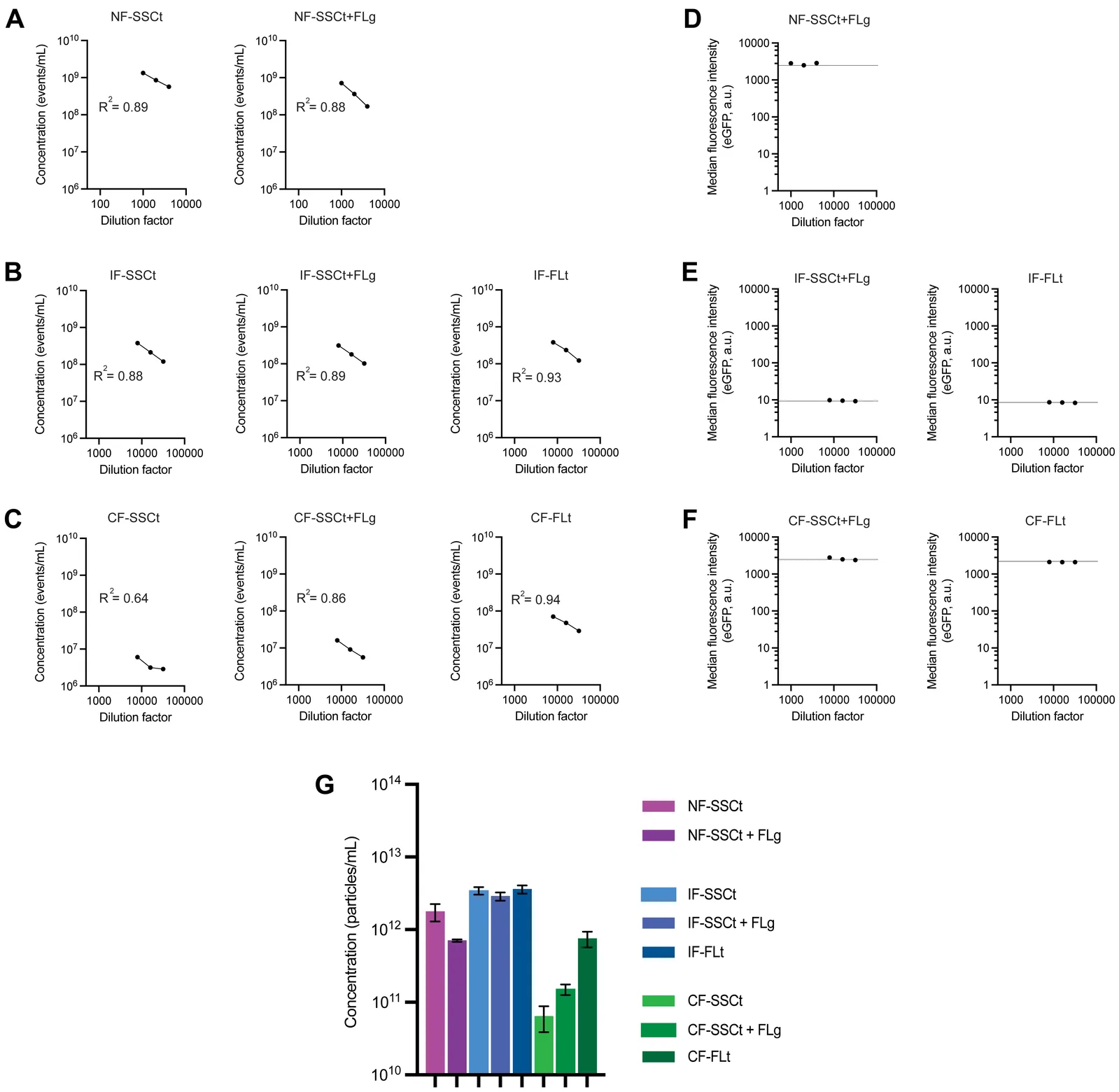
Quantitative analysis of fluorescent rEVs. (A-C) Linear regression analysis showing concentration of measured particles across three selected dilutions (1:1,000, 1:2,000, 1:4,000 for NF and 1:8,000, 1:16,000, and 1:32,000 for IF and CF). R^2^ values are indicated in each graph to show goodness-of-fit. Detected events in each sample were normalized by buffer control and flow rate according to detection strategy and each instrument (SSCt, SSCt + FLg, or FLt); (D-F) Median fluorescence intensity of gated fluorescent rEVs across three selected dilutions and different detection strategies (SSCt + FLg or FLt); (G) Bar graph of measured rEV average concentrations using the indicated three optimal dilutions across different threshold options (SSCt or FLt). For comparison, we used a FLg over the total events detected with SSCt to select for the fluorescent rEV population on the NF, IF, and CF. Concentration measurements are corrected for time, dilution factor, PBS buffer control, and flow rate for each instrument and detection mode. rSD is calculated from the selected three dilutions performed from the same sample for each detection strategy (SSCt, SSCt + FLg, or FLt). The data shown is representative of two independently performed experiments acquiring six diluted samples from the same stock on each instrument. CF: CytoFLEX LX; FL: fluorescence; FLg: fluorescence gate; FLt: fluorescence threshold; IF: BD Influx; NF: NanoFCM; PBS: phosphate-buffered saline; rEVs: recombinant extracellular vesicles; rSD: robust standard deviation; SSCt: side scatter threshold.

### Particle concentration analysis of fluorescent rEVs

For comparison of measured events present in the fluorescent rEV samples across the three instruments and detection strategies, we calculated particle concentrations (events/mL). For this purpose, we used the detected events in each diluted sample and subtracted the number of events present in the buffer control. Next, we normalized per dilution factor and flow rate on each specific instrument. This allowed us to compare detected events across the different threshold parameters and instruments [Figure 4].

### Statistical analysis

Statistical analyses were performed using Microsoft Excel and figures were prepared using GraphPad Prism version 10.0 (GraphPad Software Inc). For comparison of data across instruments, we divided our dataset in two sample dilution groups derived from the same stock of fluorescent rEV and each containing three dilution points. The dilution group for the NF contained samples 1:1,000, 1:2,000 and 1:4,000 while the dilution group for the IF and CF contained samples 1:8,000, 1:16,000 and 1:32,000. Simple linear regression analysis was performed to assess goodness-of-fit, R^2^ values above 0.80 were considered an acceptable fit for single-particle detection.

## RESULTS

### Light scatter-based thresholding analysis of a mixed population of non-fluorescent SiNPs shows differences in sensitivity across platforms

We first used the SSC detector from the blue laser (488 nm) present on all three instruments to set the threshold as a comparison point due to the absence of an FSC detector on the NF and the lack of sensitivity from the FSC detector on the CF for small particle detection. Firstly, we measured a buffer control (Milli-Q control, here used as dilution buffer) with the selected settings for EV analysis. This buffer control revealed the number and distribution of background signals after a fixed time-based measurement of 120 s across all instruments [Figure 1A-C (left)]. Next, we measured the mixed population of non-fluorescent SiNPs with increasing diameter size, including populations of 68, 91, 113, and 155 nm.

The NF detected all four populations based on SSC (488 nm) signals [Figure 1A (right)], after removing events that did not show a linear relationship between their scattering area and height intensities (SSC-H *vs*. SSC-A, Supplementary Figure 1A). Although the smallest-sized populations could not be resolved from the background events detected in the buffer control [Figure 1A (left) and F]. Similarly, events that showed a linear relationship between their scattering width and height intensities (SSC-W *vs*. SSC-H) were selected on the IF [Supplementary Figure 1B], which revealed that the SSC (488 nm) threshold on the IF [(IF-side scatter threshold (SSCt)] rendered lower sensitivity compared to the NF, as the 91 nm SiNP population was the smallest detected population when compared to the buffer control [Figure 1B]. Indicating that the 68 nm SiNP population was not discriminated from the background on the IF. When we used the SSC (488 nm) threshold on the CF (CF-SSCt), none of the four SiNP populations were resolved based on SSC and/or FSC signals from the blue laser, and the measured SiNP sample showed events similar to the buffer control [Figure 1C]. Alternatively, on both the IF and the CF, we had the possibility to set the threshold based on other light scatter parameters that were more favorable for these instruments. On the IF, we used the reduced wide-angle FSC threshold (488 nm) (IF-FSCt), and on the CF we used the Violet SSC threshold from the 405 nm laser (CF-VSSCt). When using the IF-FSCt, we observed 2.9-fold fewer events in the buffer control [Figure 1D (left)] compared to the SSC (488 nm) threshold [Figure 1B (left)], mainly due to a lower background contribution of optoelectronic components. Upon selection of linear FSC-W and FSC-H events [Supplementary Figure 1C], we confirmed an improved detection of the 91 nm SiNP population [Figure 1D (right)] compared to the SSCt [Figure 1G and H], but the smallest 68 nm SiNP population was still not detectable. The CF-VSSCt measurements showed a similar number of events in the buffer control compared to the SSC (488 nm) threshold [Figure 1C and E (left)]. However, after selecting events with a linear distribution of their violet scattering area and height intensities (VSSC-H *vs*. VSSC-A, Supplementary Figure 1D), the VSSC parameter revealed three populations in the SiNP sample [Figure 1E (right)], with the 91 nm SiNP being the smallest detected population, although it was also not separated from the background signals [Figure 1I].

To validate that the 91 nm SiNPs were detectable above the LOD on the IF and CF, the 91 nm SiNPs were measured in the absence of the other three size populations. Compared to the multi-peak sample, the same 91 nm SiNP population was consistently observed across platforms [Supplementary Figure 2]. Lastly, to illustrate the impact of materials with higher refractive indices, we measured a PSNP sample containing four populations of beads with sizes in the same range as the SiNP sample (i.e., from 64, 94, 127 to 156 nm in diameter). Using the most favorable light scattering thresholding options for each platform (namely NF-SSCt, IF-FSCt, and CF-VSSCt), we showed that all four PS bead populations could be detected on the three platforms, including the smallest 64 nm PSNPs [Supplementary Figure 3], which was expected due to the higher RI of the PS material compared to silica and subsequently to biological EVs.

Overall, these findings demonstrate that light scatter-based detection sensitivity strongly depends on both instrument configuration and thresholding strategy, with particle RI further influencing the limits of nanoparticle detection across platforms.

### Low and high sample concentration impairs robust analysis of nanoparticle subpopulations across platforms

To investigate the dynamic concentration range on the three different platforms, we prepared six dilutions of the SiNP sample containing 4 sized populations and measured with the most favorable light scattering threshold for each platform (NF-SSCt, IF-FSCt and CF-VSSCt). The most concentrated SiNP sample (1:100 dilution) was favorable on the NF, allowing to clearly visualize the four sized populations [Figure 2A (left)]. However, lower concentrations (1:4,000 and 1:8,000 dilutions) showed an impaired discrimination of the four bead populations [Figure 2A (right)]. This can be explained by particles contained in the buffer becoming more abundant relative to the particles of interest in the sample, thereby compromising discrimination of the beads overlapping the most with the background (i.e., the 68 and 91 nm populations). Importantly, using the NF-SSCt, it was possible to perform a robust single particle analysis from a stock concentration of 1E11 particles/mL that was diluted to a measuring concentration of 1E9 particles/mL (1:100 dilution). Further analysis of the linearity expected from single particle detection revealed that samples diluted by 1:500, 1:1,000, and 1:2,000 showed the highest agreement [Figure 2D] while keeping a constant median scattering intensity [Figure 2G], which is expected from measurements that do not suffer from coincidence or swarm detection. In contrast, this high measuring concentration induced swarm detection when measured on both the IF-FSCt and the CF-VSSCt [Figure 2B and C (left)]. The known characteristics of swarm detection, i.e., a non-linear relationship between the number of detected events and the dilution factor, and aberrant light scattering signals^[15]^, were clearly visible in the 1:100 and 1:500 diluted sample acquired with CF-VSSCt [Figure 2C] and to a lower extent in the 1:100 diluted sample acquired with IF-FSCt [Figure 2B]. This observation was confirmed by the assessment of quantitative signals, revealing that lower sample concentrations were preferred for the IF, as in the 1:2,000, 1:4,000, and 1:8,000 diluted samples [Figure 2E-H]. Likewise, the CF data also showed a linear relationship in the 1:1,000, 1:2,000, and 1:4,000 dilutions, while analysis of the most diluted 1:8,000 sample was obscured by the background signals [Figure 2F-I]. Moreover, the data from the concentrated samples 1:100 and 1:500 obtained with the CF and IF showed non-linear relationships, indicating nonreliable single particle detection. Collectively, our results show that low particle concentrations challenge single particle detection on the NF, while high particle concentrations compromise single particle detection on the IF and CF when light-scatter based measurements are performed.

### Qualitative and quantitative analysis of fluorescent rEVs reveals differences across detection strategies and platforms

To compare the analysis of biological EVs across the three platforms, we conducted parallel measurements using identical samples of commercially available fluorescent rEVs. These rEVs have consistent biophysicochemical properties like those of native EVs and contain encapsulated EGFP (enhanced green fluorescent protein). The EGFP allows for its detection in combination with light scattering signals, adding an extra layer of specificity that enables the discrimination of fluorescent rEVs from other particles that might be present in the preparation and the dilution buffers. Firstly, an SSC (488 nm) threshold was applied on all three platforms. Besides the rEV sample, a PBS buffer control, here used as dilution buffer, was measured under the same settings [Figure 3 (left)]. Using the NF-SSCt, fluorescent rEVs are resolved as a separate population based on the fluorescence collected in the fluorescein isothiocyanate (FITC) channel [Figure 3A], with an increased event rate of 12-fold, from 10 events/s in the buffer control to 120 events/s in the rEV sample. The IF-SSCt measurements revealed a clear population of consistently increasing light scatter and fluorescence intensities [Figure 3B (top)], with an increased event rate of 2.6-fold, from 7,483 events/s in the buffer control to 19,464 events/s in the sample. Furthermore, for the IF a secondary light scatter parameter, the rw-FSC, could be plotted *vs*. fluorescence, which revealed a better separation of the fluorescent rEVs from the background [Figure 3B bottom)]. The CF-SSCt measurements again showed a high number of events in the buffer control [Figure 3D], which translated in a minimal event rate difference between the buffer control (9,827 events/s) and the rEV sample (10,307 events/s). Furthermore, the fluorescent rEVs were also very difficult to observe in the SSC *vs*. fluorescence dot plot. However, when the more favorable VSSC parameter for the CF was plotted *vs*. fluorescence, the fluorescent rEV population was visible [Figure 3D (bottom)].

Next, we utilized the EGFP signal present in the rEV to explore fluorescence-based thresholding on the IF and the CF. Since on the NF the fluorescent rEVs are fully separated from the background [Figure 3A], a simple gate suffices to select the fluorescent population on the NF. In contrast, both the IF and CF require setting the threshold prior to acquisition and recording of the samples. Using this strategy, background events in the buffer control were strongly decreased as expected, resulting in 785-fold and 111-fold reduction, respectively, for influx fluorescence threshold (IF-FLt) and cytoflex LX fluorescence threshold (CF-FLt) measurements [Figure 3C and E (left)]. This strategy also proved not to compromise the detection of the fluorescent population of rEV [Figure 3C and E (right)], which was followed by the measurement of consecutive sample dilutions [Supplementary Figure 4].

Since EV concentration is the most reported metric in literature utilizing single particle EV analysis and has been proven relevant in health *vs.* disease conditions, we next calculated the particle concentrations of measured rEVs with either SSCt, SSCt and fluorescence gating [Scatter threshold and fluorescence gating (SSCt + FLg)] (gating strategies are shown in Supplementary Figure 5), or FLt. Due to the previously observed differences in sample concentration that yielded an optimal event rate across the three instruments' measurements of the fluorescent rEV, we selected six reciprocal dilutions of the sample (1:1,000, 1:2,000, 1:4,000, 1:8,000, 1:16,000 and 1:32,000). From these measurements, we selected for each platform the three optimal sample dilutions for single particle analysis. For the NF quantification, we used more concentrated samples (1:1,000, 1:2,000 and 1:4,000), while for the IF and CF quantifications, we used more diluted samples (1:8,000, 1:16,000, 1:32,000). Single EV detection using the selected dilutions was further confirmed by a consistent median fluorescence intensity population from the eGFP rEV signals [Figure 4D and F]. Linear regression analysis showed good linearity for all detection strategies (SSCt, SSCt + FLg and/or FLt) on the NF and IF [Figure 4A and B]. For the CF-SSCt detection, it resulted in poor linearity due to the influence of the background. However, by the use of SSCt + FLg and FLt, the linearity improved considerably (R^2^ value from 0.64 to 0.86 and 0.94, respectively) [Figure 4C].

Lastly, the three selected sample dilutions were used to calculate average concentrations and the robust standard deviation (rSD). Since the NF fully resolved the fluorescent rEV population using SSCt, fluorescent events were gated from the SSCt measurements (SSCt + FLg, as shown in Supplementary Figure 5). To correct for the observed differences between instruments in terms of events present in the dilution buffer, we subtracted those from the sample and normalized particle counts to the dilution factor and flow rate used. Quantification using the three rEV samples (1:1,000, 1:2,000 and 1:4,000) revealed 1.7E12 events/mL ± 27% (rSD). This variability was effectively reduced when a fluorescent gating was applied around the EGFP signal present in the rEV population (NF-SSCt + FLg), resulting in an average concentration of 7E11 events/mL ± 4% (rSD, Figure 4G). These findings illustrate that robust quantitative measurements can be achieved when EV samples are analyzed in an appropriate dilution and that fluorescence signals ensure reliable detection of particles of interest.

For the IF, the calculated average concentrations of rEVs measured using IF-SSCt, IF-SSCt + FLg, and IF-FLt were 3.4E12 events/mL ± 12% (rSD), 2.9E12 events/mL ± 13% (rSD) and 3.6E12 events/mL ± 13% (rSD), respectively. The great consistency across the different detection strategies on the IF can be attributed to a lower impact of background events across settings.

In contrast, the CF measured rEV concentrations showed a bigger discrepancy among the different detection strategies and dilutions, mainly caused by the high background noise on the CF. After background subtraction, the number of events in the samples drops drastically, leading to erroneous quantification and underestimation of the number of fluorescent rEVs. Background noise is lowered by applying fluorescent gating on the CF around the EGFP-expressing rEVs (SSCt + FLg), which results in improved rEV detection and a higher particle concentration, albeit to a lower extent when compared to directly applying a FLt [Figure 4G]. Measurements using the CF-SSCt, CF-SSCt + FLg, and CF-FLt with the lowest concentrated rEV samples (1:8,000, 1:16,000, and 1:32,000) resulted in an average concentration of 6.4E10 events/mL ± 39% (rSD), 1.5E11 events/mL ± 17% (rSD) and 7.5E11 events/mL ± 24% (rSD), respectively. From which the last detection strategy (CF-FLt) showed the highest agreement with the other two instruments (NF-SSCt + FLg and IF-SSCt + FLg and IF-FLt). Overall, rEV quantification based on SSC-thresholding only resulted in high differences among instruments, compared with FL-based detection (either by NF-SSCt + FLg or IF-FLt and CF-FLt). Furthermore, selecting the appropriate sample dilution per instrument is a crucial step for robust EV quantification, and fluorescence-based detection revealed the highest quantification agreement among instruments.

## DISCUSSION

The advancement of high-sensitivity FC has opened new opportunities for the multiparametric characterization of heterogeneous samples containing submicron-sized particles, including NPs and EVs. To evaluate the strengths and limitations of different instruments, it is important to perform cross-platform studies that measure identical samples and validate the utility of RM. Here, we evaluated three instruments from different generations that have reported capabilities for measuring EVs at the single particle level^[4,5,14,16]^ and followed the MIFlowCyt-EV framework for data reporting and included the use of relevant controls and well-characterized RM (SiNPs and rEVs)^[2]^. To minimize external variations due to storage and transportation of samples^[17]^, we performed the experiments with identical samples simultaneously in the same location. Furthermore, the three platforms were operated in parallel, each by an expert for the respective platform.

To compare light scatter-based measurements across platforms, we analyzed mixed populations of well-characterized SiNPs and PSNPs. While the NF was the only platform that could detect the smallest population of 68 nm non-fluorescent SiNPs, our results show that at low sample concentrations the detection of the smallest SiNP populations (68 and 91 nm) by the NF was impaired due to the overlap with background noise. Both the IF and CF were able to detect the second smallest SiNP population (91 nm), but they required a 10-fold lower sample concentration compared to the NF to ensure single particle detection, which partially accounts for differences in the sample flow rate among the three instruments. Importantly, the high background signals of the CF obscured SiNP analysis to a much greater extent compared to the IF. The latter has much lower background interference and two high-sensitivity light scatter parameters. Overall, the background noise can substantially compromise light-scatter analysis of NPs and EVs, indicating a preference for instruments with low background signal when small particles are measured.

Furthermore, we here demonstrate clear differences in the preferred light-scatter triggering strategy between the three platforms, i.e., NF-SSCt, IF-FSCt, and CF-VSSCt, showing the best discrimination between background signals present in the dilution buffer and NP samples. This illustrates that there is no ‘‘one-fits-all’’ strategy that can be applied to all flow cytometers, but that the choice depends on the design and configuration of the different platforms, as well as the samples that are being measured. Here, we selected a SSC thresholding strategy (488 nm) for comparison purposes across platforms, as it was the only light scatter-based thresholding option available on all platforms. FSC thresholding (488 nm) was only possible on the IF, as the NF completely lacks the FSC and the CF lacks the required FSC sensitivity. Likewise, VSSC thresholding (405 nm) was only available on the CF. Interestingly, our measurements revealed that the optimized scatter-based thresholding strategy for each platform (namely NF-SSCt, IF-FSCt and CF-VSSCt) exhibited sufficient light-scatter sensitivity to detect SiNPs as small as 91 nm and to discriminate distinct subpopulations that differ by only 23 nm in size (113 nm *vs*. 91 nm). In addition, the results show the added value of mixed reference SiNP populations to investigate the performance of high-sensitivity flow cytometers in relation to single EV analysis. Since silica has a much lower RI compared to PS, silica is preferred when low-scattering particles, such as EVs, will be analyzed^[3]^. Such mixed-reference SiNP populations (up to 500 nm in size) are also of great interest for calibration and standardization of other orthogonal single NP and EV analysis technologies^[18]^.

However, since many published articles on EV flow cytometric analysis still refer to PS beads as reference, we also included PSNPs in our analysis and show that 64 nm PSNP could be detected on the three platforms using the selected thresholding strategies.

Besides synthetic NPs, tailored biological RM have been proposed as helpful tools to assess instrument performance in relation to EV analysis. Here, we took advantage of commercially available fluorescent rEVs^[13]^ and showed that these rEVs with an average size of 125 nm could be detected on all three platforms, thereby providing a valuable tool for instrument assessment^[13]^. In addition, using fluorescent rEVs, we evaluated their detection using fluorescence thresholding, which effectively reduced background noise. Moreover, combining light scatter thresholding with fluorescence analysis of these rEVs enabled us to circumvent the impact of undesired background signals, thereby improving the robustness of rEV quantification.

Importantly, we here demonstrate that sample concentration severely impacts both qualitative and quantitative analysis of fluorescent rEVs across flow cytometers. Consistent with our SiNP data, we found that the NF with a 0.005 µL/min sample flow rate required at least a 10-fold higher sample concentration (E9 particles/mL) compared to the IF, with a 6 µL/min sample flow rate, and the CF, with a 10 µL/min sample flow rate (E8 particles/mL). We showed that low-concentration samples can be measured on the NF, but that SSC-based detection resulted in accumulation of background events, causing an overestimation of the rEV concentration. On the other hand, the higher rEV concentrations, optimal for the NF, introduced swarm detection (massive coincidence) on the IF and CF, thereby compromising quantitative analysis^[1,15,19]^. Based on these findings, we emphasize that for biological EV samples of unknown concentration, sample dilutions are indispensable and need to be adjusted and validated for each instrument^[2]^. Besides the reference synthetic NPs that are commonly used for instrument assessment, we therefore advocate the use of well-characterized fluorescent rEVs as a suitable reference material to evaluate and compare EV detection capabilities across different platforms, allowing to investigate both light scatter and fluorescence-based detection. With our current setup, we were able to investigate sample concentration concordance across the three systems and found that fluorescence-based detection greatly improved the agreement of measured concentration. However, it is important to note that concordance does not imply accuracy. High concordance only indicates that measurements obtained using different instruments agree with one another; it does demonstrate that the instruments are measuring the true concentration.

Previously, the added value of calibration using commercial materials such as fluorescence reference beads with molecules of equivalent soluble fluorophore (MESF) units and non-fluorescent National Institute of Standards and Technology (NIST)-traceable beads for both light-scatter and fluorescence flow cytometric EV analysis has been clearly indicated, and specific modelling software, i.e. FCM PASS, has been developed^[3,20]^. However, using these calibration strategies for the comparison of instruments from different generations is not so straightforward. The older generation of instruments with analogue detection, such as the IF used in this study, cannot be calibrated in this way due to the lack of precise signal digitalization^[21]^. Furthermore, the light scatter detection range of the NF does not allow for measuring PS particles bigger than 500 nm in size with the settings required for small EV analysis, thereby hampering the utilization of the available tools to be implemented for studies such as the one presented here.

A limitation of this study was the inability to implement and harmonise scatter and fluorescence instrument calibration across all three instruments.

Future studies would benefit from the use of calibration materials that are compatible across instruments from different generations so that results can be presented in standardized units rather than arbitrary units. The implementation of small and dim calibration materials with assigned uncertainty values and an RI close to EVs would enable the definition of the LOD on each platform, which ensures robust data interpretation by defining the measured concentrations across a detection range. This is important since the smaller fraction of EVs under 100 nm diameter might not be detectable across all instruments, which will impair accurate particle quantification^[22]^.

In addition, our study did not assess the quantitative accuracy of particle concentration measurements. Although RM were included, these materials are not certified standards with NIST-traceable concentration values and do not provide uncertainty estimates for the assigned concentrations. For example, the silica nanoparticle RM have reported particle sizes but not certified concentrations for the individual populations. Similarly, the concentration of the recombinant EV stock was originally determined by NTA, a method known to yield systematically different concentration estimates compared with high-sensitivity FC. Consequently, the available concentration values cannot be considered definitive ground-truth measurements for accuracy assessment. For this reason, we did not attempt to identify any instrument as the most accurate or compare measured concentrations against nominal values. Instead, the focus of this study was the comparative evaluation of measurements across instruments and detection strategies. Future studies aimed at assessing quantitative accuracy should employ certified RM with traceable concentration values and associated uncertainty estimates, ideally using other orthogonal technologies than high-sensitivity FC to further address concordance and accuracy of particle concentrations^[23]^.

Furthermore, while we focused on the comparative evaluation of the same reference samples across three platforms, future studies could systematically evaluate intra-instrument and inter-instrument variability by comparing the performance of multiple instruments representative for each platform, within the same laboratory setting. In addition, evaluating inter-laboratory variability remains important to ensure robust detection of EV signatures^[24]^ and to establish concentration measurements with acceptable variability across different platforms^[25]^.

To further advance cross-platform studies and ultimately ease the translation potential of EVs into the clinic, future developments are needed to expand the current portfolio of calibrators in the nanometric range, aiming to be compatible across flow cytometric instruments including the newest generation of fully dedicated high-sensitivity analyzers, and orthogonal approaches for NP analysis^[26]^.

In conclusion, our comparative study provides a comprehensive evaluation of three high-sensitivity flow cytometers from different generations. Our findings highlight platform-specific differences in sensitivity, optimal sample concentration, and scatter- or fluorescence-based detection strategies for the characterization of nanoparticles and extracellular vesicles. These findings may also provide a foundation for the selection and optimization of flow cytometry approaches in future studies investigating small particles and EVs in complex biological samples.

## DECLARATIONS

### Availability of data and materials

The flow cytometry datasets generated during this study are available in the Figshare dataset repository https://doi.org/10.6084/m9.figshare.32323827.

### AI and AI-assisted tools statement

During the preparation of this manuscript, the AI tool ChatGPT (version 5.5, released 2025-08-07) was used for generating elements presented in the graphic abstract such as the instrument representations and the EV illustration. The tool did not influence the study design, data collection, analysis, interpretation, or the scientific content of the work. All authors take full responsibility for the accuracy, integrity, and final content of the manuscript.

### Copyright

© The Author(s) 2026.
