# Supplementary material for: Characterization of nanoparticles and fluorescent recombinant extracellular vesicles using three different generations of high-sensitivity flow cytometers
Source: Extracell Vesicles Circ Nucl Acids. 2026 Aug 18;7(3):1314–30. doi: 10.20517/evcna.2026.33 (PMC13678172; doi:10.20517/evcna.2026.33)
Supplement: Supplementary file 1 [file evcna-7-3-1314-SupplementaryMaterials.pdf]

Supplementary Materials

Characterization of nanoparticles and fluorescent recombinant extracellular vesicles using three different generations of high-sensitivity flow cytometers

Estefanía Lozano-Andrés[1,2], Ye Tian[3], Sten F.W.M. Libregts[1], An Hendrix[4,5], Xiaomei Yan[3], Ger J.A. Arkesteijn[1,2], Marca H.M. Wauben[1]

[1]Division of Cell Biology, Metabolism and Cancer, Department of Biomolecular Health Sciences, Faculty of Veterinary Medicine, Utrecht University, Utrecht 3584 CM, The Netherlands.

[2]Division of Infection & Immunology, Department of Biomolecular Health Sciences, Faculty of Veterinary Medicine, Utrecht University, Utrecht 3584 CL, The Netherlands.

[3]MOE Key Laboratory of Spectrochemical Analysis & Instrumentation, Key Laboratory for Chemical Biology of Fujian Province, State Key Laboratory of Physical Chemistry of Solid Surfaces, Department of Chemical Biology, College of Chemistry and Chemical Engineering, Xiamen University, Xiamen 361005, Fujian, China.

[4]Laboratory of Experimental Cancer Research, Department of Human Structure and Repair, Ghent University, Ghent 9000, Belgium.

[5]Cancer Research Institute Ghent, Ghent 9000, Belgium.

Correspondence to: Dr. Estefanía Lozano-Andrés, Division of Cell Biology, Metabolism and Cancer, Department of Biomolecular Health Sciences, Faculty of Veterinary Medicine, Utrecht University, Utrecht 3584 CM, The Netherlands. E-mail: e.lozanoandres@uu.nl

Supplementary Material & Methods

Polystyrene Nanoparticles (PSNPs) of different sizes (64, 94, 127 and 156 nm in diameter) were purchased from Bangs Laboratories, Inc. These PSNPs were prepared into a mixture and diluted with Milli-Q H_2O (filtered 0.22 µm, Merck) water prior to the measurements.

Supplementary Table 1. Author checklist: MIFlowCyt-compliant items

**Requirement**	**Please include requested information**
1.1. Purpose	The purpose of this study is to evaluate the performance of three different flow cytometers when measuring silica nanoparticles (SiNP) and recombinant fluorescent Extracellular Vesicles (rEV)
1.2. Keywords	extracellular vesicles, exosomes, high-sensitivity, flow cytometry, silica nanoparticles, characterization, cross-platform, reference material
1.3. Experiment variables	Sample dilution (as indicated) and detection strategies
1.4. Organization name and address	Department of Biomolecular Health Sciences<br>Faculty of Veterinary Sciences<br>Utrecht University<br>Yalelaan 2, 3584 CM<br>Utrecht, The Netherlands
1.5. Primary contact name and email address	Dr. Estefanía Lozano Andrés<br>e.lozanoandres@uu.nl
1.6. Date or time period of experiment	January 2019
1.7. Conclusions	We provide information on the performance of three different high-sensitivity flow cytometers to characterize nanoparticles and EVs
1.8. Quality control measures	Dilution buffer control<br>Serial dilutions

2.1.1.1. (2.1.2.1., 2.1.3.1.) Sample description	Silica Nanoparticles and rEV
2.1.1.2. Biological sample source description	Described under material & methods
2.1.1.3. Biological sample source organism description	Human
2.1.2.2. Environmental sample location	
2.3. Sample treatment description	N/A
2.4. Fluorescence reagent(s) description	N/A
3.1. Instrument manufacturer	NanoFCM, Becton Dickinson and Beckman Coulter
3.2. Instrument model	NanoFCM N30 model<br>BD Influx™ optimized instrument for detection of sub-micron sized particles as described previously<br>CytoFLEX LX
3.3. Instrument configuration and settings	BD Influx optimized to measure small particles. All configuration details can be found in van der Vlist, E. J., Nolte-'t Hoen, E. N., Stoorvogel, W., Arkesteijn, G. J., and Wauben, M. H. (2012) Fluorescent labeling of nano-sized vesicles released by cells and subsequent quantitative and qualitative analysis by high-resolution flow cytometry. Nat Protoc 7, 1311-1326 and Arkesteijn, G.J.A., et al., Improved Flow Cytometric Light Scatter Detection of Submicron-Sized

	Particles by Reduction of Optical Background Signals. Cytometry A, 2020. **97**(6): p. 610-619<br>Briefly, samples were measured at a constant flow rate for 120 seconds using indicated settings (SSC, rw-FSC or FL)
4.1. List-mode data files	Are available upon request
4.2. Compensation description	No compensation was required since only one fluorescence signal was read
4.3. Data transformation details	No data transformation was applied
4.4.1. Gate description	Gates were applied based on the controls (shown in Supplementary Material & Methods)
4.4.2. Gate statistics	The number of total events recorded in 120 seconds measurements are shown in the dot plots
4.4.3. Gate boundaries	Images of the ungated and gated plots can be found in figures and supplementary data

Supplementary Table 2. MIFlowCyt-EV framework

1.1 Preanalytical variables conforming to MISEV guidelines	Yes, all relevant data can be found in provided references
1.2 Experimental design according to MIFlowCyt guidelines	Yes, MIFlowCyt checklist can be found as part of the supporting information of this manuscript
2.1 Sample staining details	Sample staining was not performed
2.2 Sample washing details	No sample washing was performed
2.3 Sample dilution details	Yes, described in the text and indicated in the figures
3.1 Buffer-only controls	Yes, relevant buffer controls were measure and are shown
3.2 Buffer with reagent controls	Not required as there was no sample staining performed

3.3 Unstained controls	Not required as there was no sample staining performed
3.4 Isotype controls	Not required as there was no sample staining performed
3.5 Single-stained controls	Not required as there was no sample staining performed
3.6 Procedural controls	Not required as there was no sample staining performed
3.7 Serial dilutions	Yes, serial dilutions were performed and are shown for SiNP and rEV samples. See Figure 2 and Supplementary Figure 3
3.8 Detergent-treated controls	Detergent treatment was not performed
4.1 Trigger channel(s) and threshold(s)	On the NF we used a SSC threshold (488 nm laser) with 40 mW and 10% of SSC decay, following the manufacturer's instructions<br>On the IF we used a SSC threshold (488 nm laser at 0.32, a.u.), rw-FSC (488 nm laser at 0.29, a.u.) and FL (488 nm laser at 0.61, a.u.)<br>On the CytoFLEX LX we used a SSC threshold (488 nm laser at 500, a.u.), VSSC threshold (405 nm laser at 1600 a.u.) and FL threshold (488 nm laser at 600 a.u.)
4.2 Flow rate/volumetric quantification	Yes, low flow rate was kept constant across settings for each instrument. Flow rate on the NF was 0.005 µL/min, on the IF was 6 µL/min and on the CF was 10 µL/min
4.3 Fluorescence calibration	Not performed due to lack of materials available compatible with all instruments at the time of the study
4.4 Scatter calibration	N/A
5.1 EV diameter/surface area/volume approximation	N/A

5.2 EV refractive index approximation	N/A
5.3 EV epitope number approximation	N/A
6.1 Completion of MIFlowCyt checklist	Yes, see Table S1
6.2 Calibrated channel detection range	N/A
6.3 EV number/concentration	Yes, see Figure 4
6.4 EV brightness	N/A
7.1 Sharing of data to a public repository	All data files are available upon request

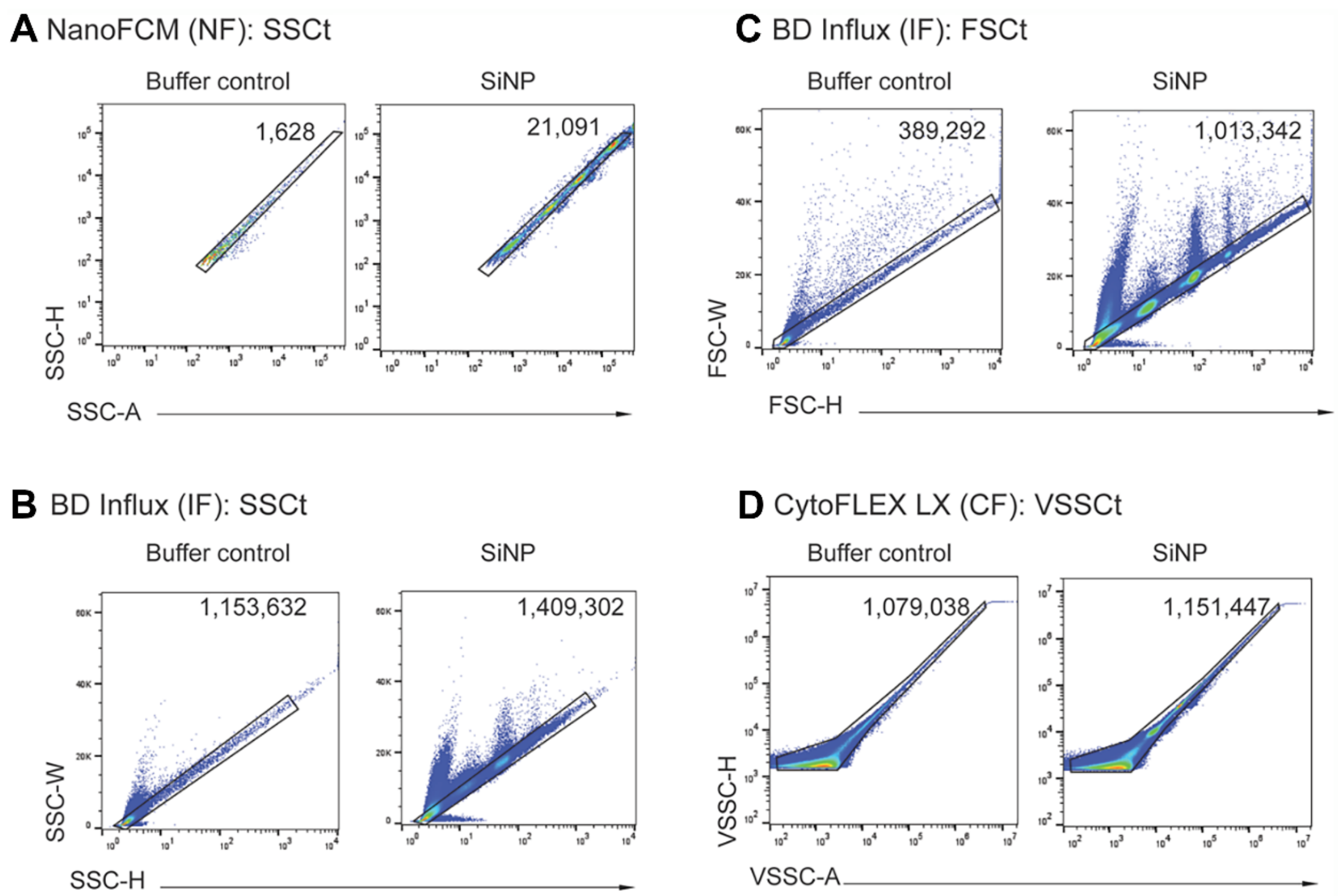

Supplementary Figure 1. Buffer control and Silica nanoparticles measured across instruments with the indicated threshold combinations on (A) NF-SSCt, (B) IF-SSCt (C) IF-FSCt and (D) CF-VSSCt. Dot plots showing the gate around populations displaying a linear relationship between height (H), area (A) and/or width (W) for each indicated parameter. Number of total detected events without any background subtraction is shown in the top right corner of each dot plot. These gates allowed us to improve the selection for single particles and avoid the inclusion of some aggregates present in the sample for further analysis.

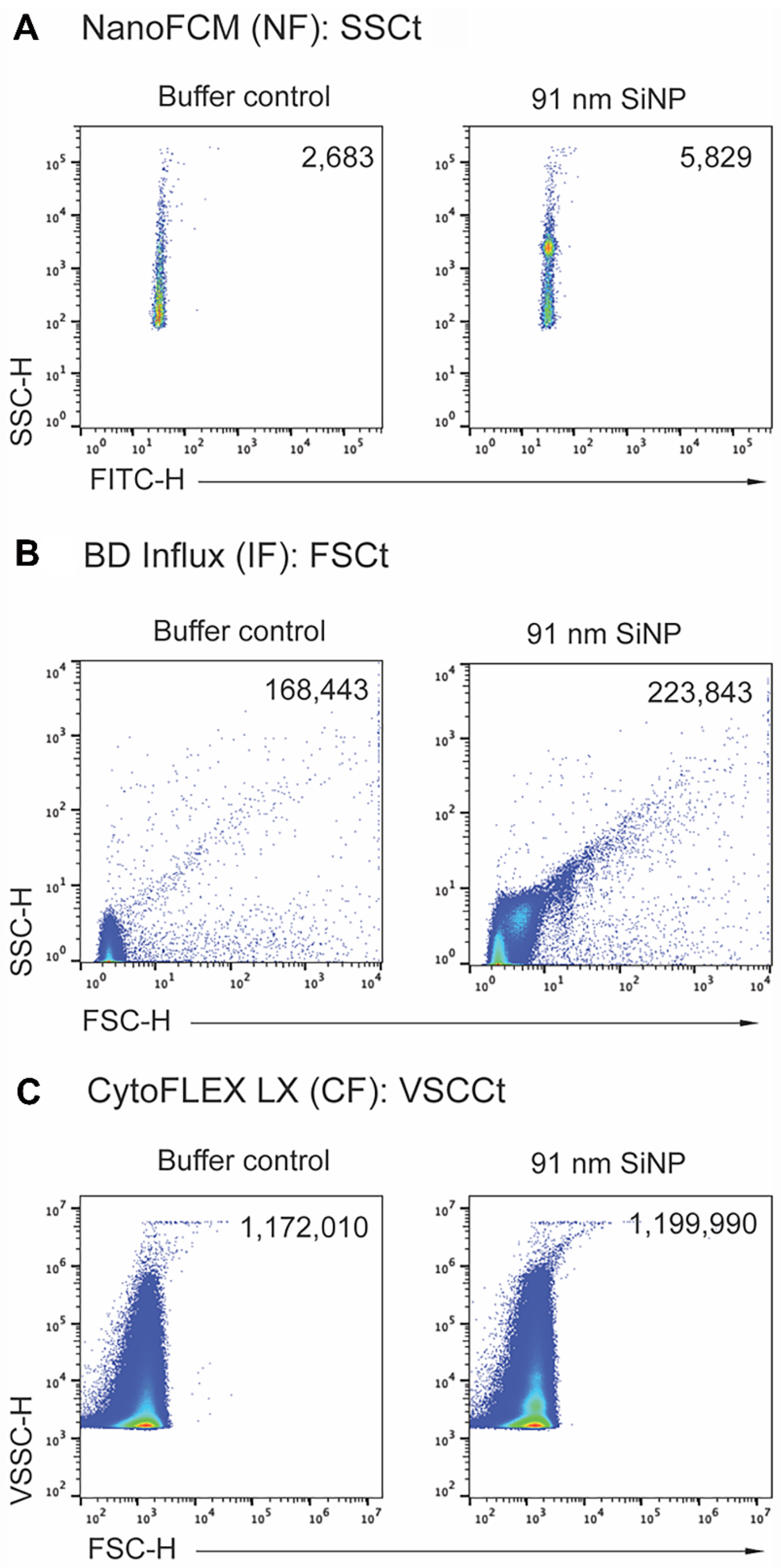

Supplementary Figure 2. Buffer control and Silica nanoparticles of 91 nm diameter were measured across instruments with the selected threshold combinations on (A) NF-SSCt, (B) IF-FSCt and (C) CF-VSSCt. Dot plots showing the selected scatter parameter (either SSC-H or VSSC-H, as indicated) Vs either FITC-H on the NF or FSC-H on both the IF and CF. Number of total detected events without any background subtraction is shown in the top right corner of each dot plot. The figure shows data obtained from one selected sample dilution measured on each instrument. In total, six diluted samples originating from the same stock solution were measured across each platform.

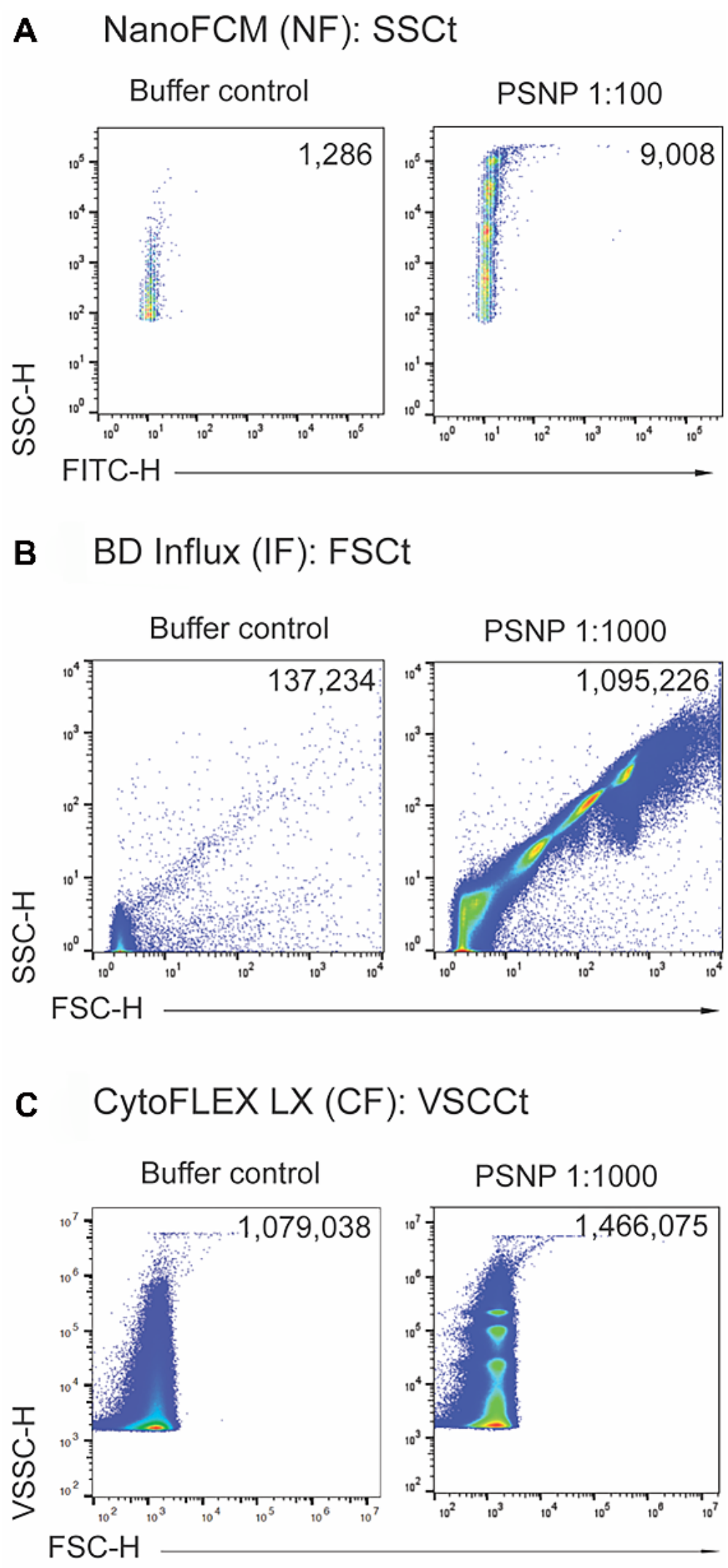

Supplementary Figure 3. Light scatter-based analysis of buffer control and a mixed population of non-fluorescent polystyrene nanoparticles across platforms. Dot plots displaying number of events as indicated in buffer control and the sample containing 4 sized populations of non-fluorescent polystyrene nanoparticles (64, 94, 127 and 156 nm in diameter). Samples were measured on the NanoFCM with (A) a SSC (488 nm) threshold, on the BD Influx with (B) a rw-FSC (488 nm) threshold and on the CytoFLEX LX with (C) a VSSC (405 nm) threshold. PSNP samples were pre-diluted shortly before measurements (1:100 for NF and 1:1000 for both IF and CF). Identical buffer control and PSNP samples were acquired simultaneously on all three instruments in the same room under the same conditions. Number of total detected events without any background subtraction is shown in the top right corner of each dot plot. The figure shows data obtained from one selected sample dilution measured on each instrument. In total, six diluted samples originating from the same stock solution were measured across each platform.

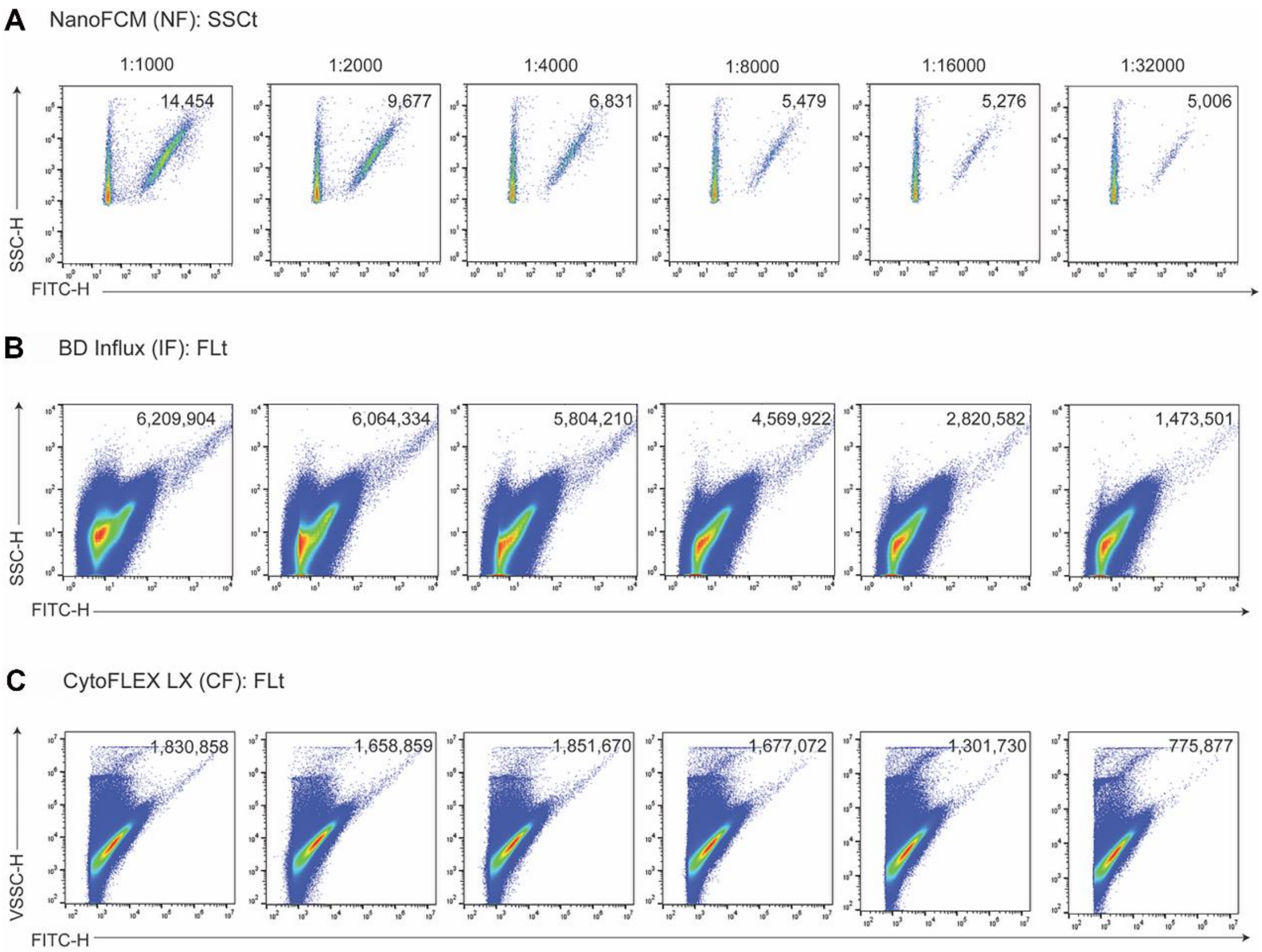

Supplementary Figure 4. Serial dilutions performed across the three platforms to define single EV detection. Dot plots showing the selected scatter parameter (either SSC-H or VSSC-H, as indicated) Vs fluorescence from the eGFP rEV samples (FITC-H). Six dilutions from the same rEV stock sample are measured on the (A) NF-SSCt, (B) IF-FLt and (C) CF-FLt. Dilution factor used for each dot plot is indicated in the bottom row, from 1:1000 to 1:32000. Matching buffer controls are shown in the main text (Figure 3A, C and E). Number of total detected events without any background subtraction is shown in the top right corner of each dot plot. The experiment shown is representative of two independently performed experiments acquiring six diluted samples from the same stock on each instrument.

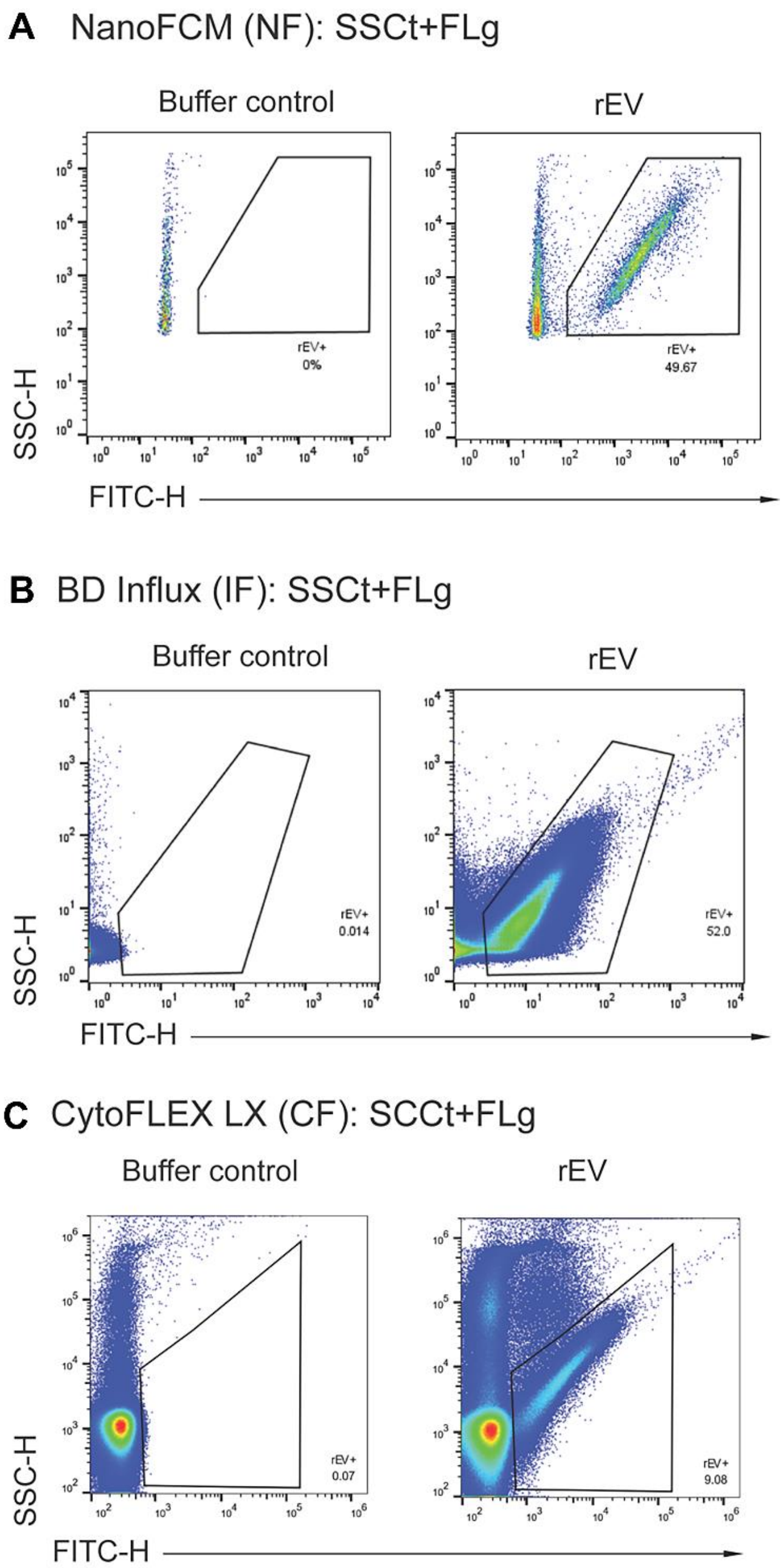

Supplementary Figure 5. Gating strategy for fluorescent rEV samples measured with SSCt. Dot plots showing the representative gate based on SSC-H Vs fluorescence from the eGFP rEV samples (FITC-H) on the (A) NF-SSCt+FLg, (B) IF-SSCt+FLg and (C) CF-SSCt+FLg from the buffer control and the rEV sample. These gates were used for the analysis shown in the main text Figure 4, representative dot plots correspond to a more concentrated sample on the NF (dilution 1:1000), and more diluted samples on both the IF and CF (dilution 1:32000). Data shown is representative of at least two independent experiments performed with six sample dilutions each for each instrument.
